# A Rare Case of Granulomatosis With Polyangiitis Complicated by Splenic Rupture

**DOI:** 10.7759/cureus.52536

**Published:** 2024-01-18

**Authors:** Olfat M Awad, Ibrahim A Srour, Fatima A Mekdad, Samih M Hamadeh, Majdi S Hamadeh

**Affiliations:** 1 Department of Nephrology, Lebanese University Faculty of Medicine, Beirut, LBN; 2 Emergency Department, Al-Zahraa Hospital University Medical Center, Beirut, LBN; 3 General Medicine, Wayne State University School of Medicine, Michigan, USA; 4 Department of Nephrology, Al-Zahraa Hospital University Medical Center, Beirut, LBN

**Keywords:** autoimmune, renal failure, rpgn, glomerulonephritis, vasculitis, biopsy, splenic rupture, aav, anca, wegener's granulomatosis

## Abstract

Granulomatosis with polyangiitis (GPA), formerly known as Wegener's granulomatosis (WG), is a condition marked by necrotizing vasculitis of the small-medium vessels that results in necrotizing granulomatous inflammation. Splenic involvement in GPA is a potentially life-threatening consequence of connective tissue disease and is rarely described as the main presenting feature. We present a case of a patient with perinuclear anti-neutrophil cytoplasmic antibodies (p-ANCA) who experienced spontaneous splenic rupture. A CT scan of the abdomen, an ANCA test, and a splenic biopsy were employed to identify ANCA-associated vasculitis (AAV) splenic rupture. Our patient's splenic rupture could be attributed to GPA. Nonetheless, since it may alter patient follow-up and therapy, a patient with spontaneous splenic rupture without an obvious explanation should be promptly evaluated for connective-tissue disease. This report highlights the intricacy and unpredictability of the clinical symptoms linked to AAV, as well as the possibility of misinterpreting them.

## Introduction

Anti-neutrophil cytoplasmic antibody (ANCA)-associated vasculitis (AAV) is an autoimmune disease that causes inflammation in the small and medium blood vessels in the presence of antibodies that target the neutrophil antigens proteinase 3 (PR3) or cytoplasmic anti-neutrophil cytoplasmic antibody (c-ANCA) and myeloperoxidase (MPO) or perinuclear anti-neutrophil cytoplasmic antibodies (p-ANCA). It is associated with a variety of symptoms depending on the organ or part of the body involved. Granulomatosis with polyangiitis [GPA; previously known as Wegener granulomatosis (WG)], eosinophilic granulomatosis with polyangiitis (EGPA; originally referred to as Churg-Strauss syndrome), and microscopic polyangiitis (MPA) are the three primary disorders that constitute this group [1,2].

GPA is a rare type of vasculitis that affects the upper respiratory tract, lungs, and kidneys and is characterized by a significant inflammatory response [3]; it is seldom reported to affect the spleen. Vasculitis, fibrinoid blood vessel alterations, necrotizing granulomatous inflammation, and infarction are some of the pathologic splenic changes linked to GPA [4]. Spontaneous splenic rupture can develop as a consequence of GPA; however, it is exceedingly rare, and only four such cases have been published in the literature [5-8]. Due to its rarity, early and accurate detection coupled with vigorous therapy is necessary to slow the progression of the disease. We discuss a case of spontaneous splenic rapture of rapidly progressive glomerulonephritis (RPGN) with p-ANCA-associated GPA, which marks the fifth reported case of spontaneous splenic rupture in association with AAV.

## Case presentation

A 58-year-old woman, with a past medical history of hypothyroidism treated with levothyroxine 100 mcg per day, presented to the Emergency Department (ED) of the Al-Zahraa Hospital in Beirut, Lebanon on February 2023, with complaints of chest discomfort, dyspnea, fatigue, and pallor. She also mentioned her lack of urine output for the past two days. Her history went back to one month before the presentation when she had experienced a flu-like illness with waxing and waning fevers, myalgia, and arthralgia for which symptomatic treatment had been prescribed by her primary physician. The patient denied any history of trauma or travel. 

On presentation to the ED, her vital signs were relevant for tachycardia of 105 beats per minute and blood pressure of 98/60 mmHg. She was afebrile with normal oxygen saturation. She had pale skin as well as a poorly injected conjunctiva. She had bilateral lung crepitations, moist mucous membranes, a soft and non-tender abdomen, no hepatosplenomegaly, and edema in the lower limb (Grade +2). Abdominal CT showed normal size, position, and density of both kidneys and ruled out obstructive uropathy. Other intra-abdominal organs, including the spleen, were also unremarkable on the first day of presentation. Pertinent initial laboratory parameters are presented in Table 1.

**Table 1:** Pertinent laboratory parameters upon presentation

Parameter	Result	Reference range
Urine analysis	Not performed	-
Blood urea nitrogen (BUN)	94 mg/dL	7-20 mg/dL
Haemoglobin (Hgb)	7.7 g/dL	12-15.5 g/dL
Creatinine	8.7 mg/dL	0.5-0.9 mg/dL
Serum sodium	135 mmol/L	135-145 mmol/L
Serum potassium	5.8 mEq/L	3.7-5.3 mEq/L
Serum bicarbonate	10 mmol/L	22-26 mmol/L
Lactate dehydrogenase (LDH)	330 IU/L	125-220 IU/L
Haptoglobin	62 mg/dL	30-200 mg/dL
Retic %	3.4%	0.2-2%
Direct bilirubin	0.2 mg/dL	0-0.5 mg/dL
Total bilirubin	0.35 mg/dL	0.2-1.3 mg/dL

**Figure 1:**
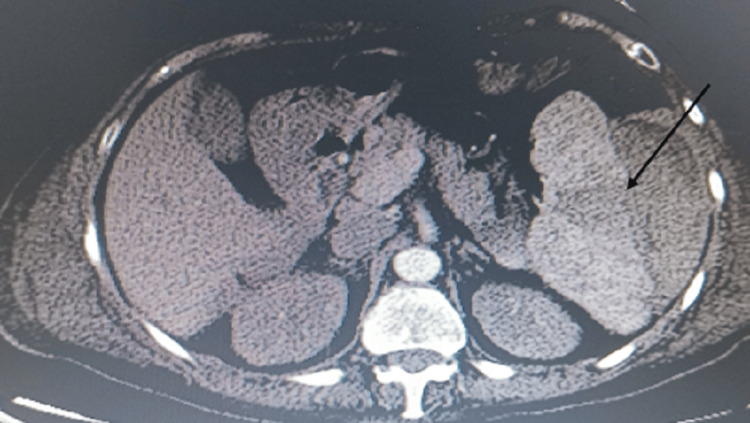
CT scan of the abdomen and pelvis The image shows an effusion of the part of the spleen (black arrow), consistent with splenic rupture CT: computed tomography

The patient was admitted to the nephrology service, started on hemodialysis, and an autoimmune workup was ordered. On the next day, she had severe abdominal pain localized to the left upper quadrant, and a hemoglobin drop to 6.5 g/dL was observed. An abdominal and pelvic contrast-enhanced CT scan demonstrated a splenic rupture with a subcapsular hematoma measuring approximately 11 x 4 cm, hyper-dense fluid surrounding the spleen, and some fluid surrounding the liver and in the pouch of Douglas (Figure 1). She was rushed to the operating room; an emergent splenectomy was performed and the sample was sent for pathology.

The patient again denied any history of any trauma to the abdomen upon further questioning. By that time, the ordered serological test results were obtained, which are detailed in Table 2.

**Table 2:** Hepatitis, HIV, and autoimmune workup serology HIV: human immunodeficiency virus

Parameter	Result	Reference range
HIV antibodies	Negative	Negative
Hepatitis C antibodies	Negative	Negative
Hepatitis B surface antigen	Negative	Negative
C3 and C4 complements	Normal	Normal
Antinuclear antibodies (ANA)	Negative	Negative
Anti–glomerular basement membrane (anti-GBM)	Negative	Negative
Cytoplasmic ANCA (c-ANCA)	Negative	Negative
Perinuclear ANCA (p-ANCA)	Positive	Negative

**Figure 2:**
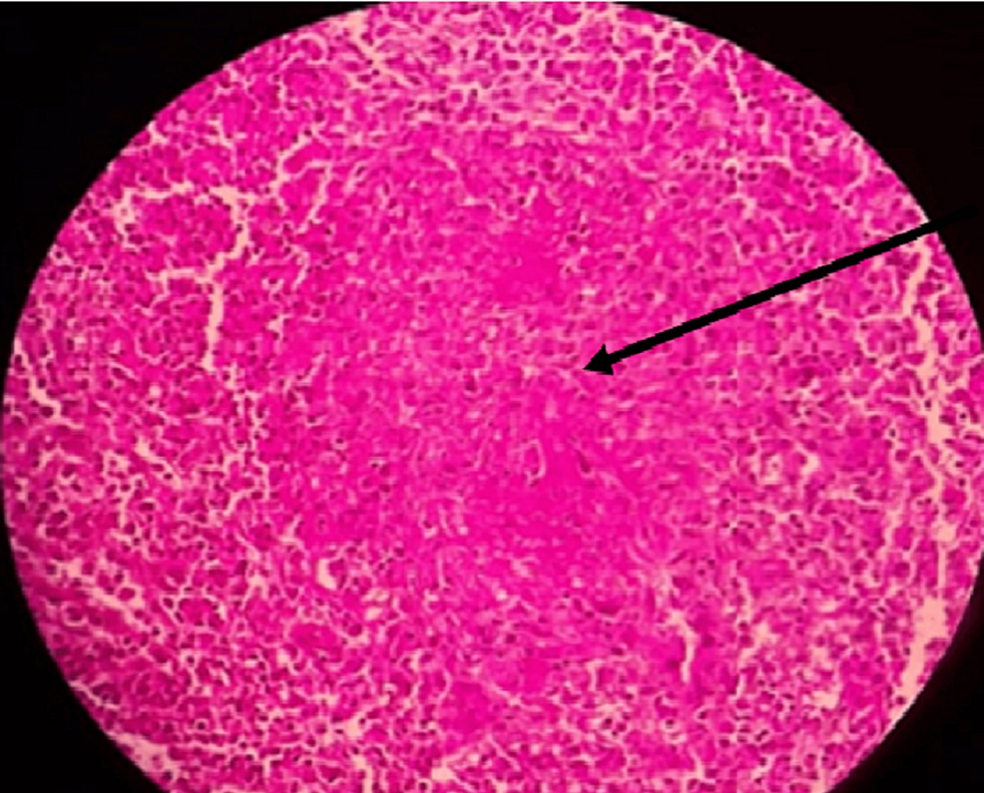
Section of the spleen showing necrotizing caseating granuloma (black arrow)

**Figure 3:**
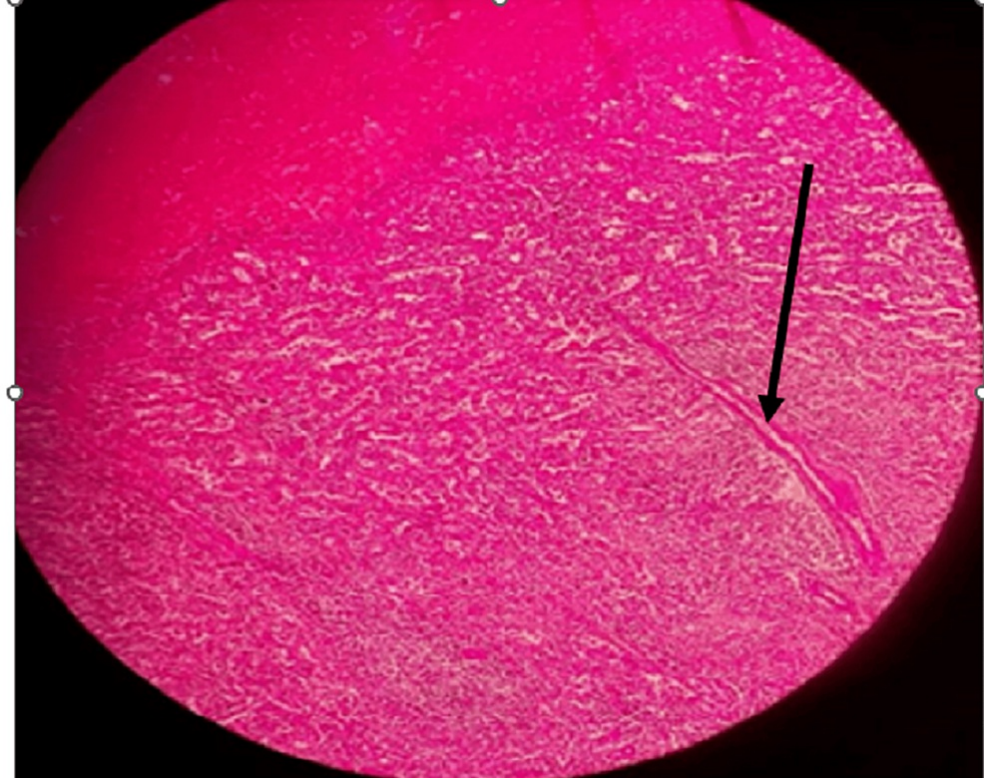
Section of the spleen showing inflammation in small to medium vessels (black arrow)

The main pathology identified on spleen biopsy involved the presence of several necrotizing granulomas (Figure 2) with diffuse mild chronic inflammation, and a few medium-size-artery vasculitis were identified (Figure 3), which confirmed the diagnosis of spontaneous splenic infarction in GPA. Therefore, severe necrosis caused by splenic infarction likely contributed to the rupture in our instance.

This patient was diagnosed as having p-ANCA-associated GPA with spontaneous (non-traumatic) splenic rupture, and she was treated with 1 mg/kg of prednisone and intravenous cyclophosphamide starting at 750 mg intravenously every three weeks. After three months of treatment, with a total dose of cyclophosphamide of 2250 mg, the patient had partial recovery of her kidney function, became dialysis-independent, and her serum creatinine level stabilized at 3 mg/dL. At this point, cyclophosphamide was stopped and a slow steroid taper was initiated, by decreasing 5 mg of prednisone every five days till it reached 5 mg per day, with close monitoring of the renal function. The patient had regular follow-ups with clinical examinations and serum creatinine tests weekly for the first month and then every month afterward.

## Discussion

Splenic rupture is a life-threatening diagnostic and therapeutic emergency and is associated with a high risk of mortality [9]. Spontaneous (non-traumatic) splenic rupture is a rare entity, and it is caused by iatrogenic, idiopathic, and myeloproliferative diseases, vasculitis, hematological malignancies, and infections (infectious mononucleosis and malaria) [10].

Spontaneous splenic rupture is an extremely rare complication of systemic vasculitis, and there have been only four reported cases of spontaneous splenic rupture in GPA patients worldwide [5,6,7,8]. In three of those cases [reported by Hawley et al. (1996), Franssen et al. (1993), and McCain et al. (2002)], the initial sign of a GPA was a splenic rupture. This contrasts with the other study conducted by Nagasu et al. (2013), where splenic rapture occurred following the diagnosis of GPA and during corticosteroid treatment. In our case, the splenic rupture occurred before diagnosis and treatment. The mechanisms of splenic rupture in three reports were different [6,7,8]; two showed no signs of vasculitis or infarction but rather a neutrophilic infiltration at the rupture site [6,8]. Another study exhibited severe necrosis but no histological signs of vasculitis [7]. However, our case is similar to the Hawley case [5], where a biopsy showed necrosis and central arteritis in the resected spleen.

Isolated cases of splenic rupture also have been noted in rheumatoid arthritis, systemic lupus erythematosus, polyarteritis nodosa [8], and EGPA [11]. The fact that GPA is more commonly associated with c-ANCA antibodies (80-90%) rather than p-ANCA antibodies (10-20%) makes our case an uncommon presentation of GPA [12]. Our patient is the fifth case of spontaneous splenic rupture in association with AAV, specifically GPA, to be reported in the literature. Early evaluation for connective tissue disease in a patient with spontaneous splenic rupture without apparent cause can significantly aid with patient follow-up and treatment [8]. The absence of a history of trauma may cause delays in identifying this life-threatening condition as the cause of a patient’s anemia or hemodynamic instability. Thus, a high index of suspicion, prompt recognition, early diagnosis by appropriate imaging, and splenectomy are essential for a favorable outcome in these patients.

## Conclusions

Spontaneous splenic rupture can complicate hematological malignancies, some infections, and vasculitides in rare cases. We discussed a case of a 58-year-old female patient diagnosed with p-ANCA-associated granulomatosis with polyangiitis complicated by spontaneous splenic rupture, a phenomenon rarely reported in the literature. This case emphasizes the importance of maintaining a high index of suspicion for spontaneous splenic rupture complicating vasculitides, which considerably changes the patient's management and needs life-saving intervention.
